# Role of serotonergic mechanism in gastric contractions induced by Indian Red Scorpion *(Mesobuthus tamulus)* venom

**DOI:** 10.4103/0253-7613.59923

**Published:** 2009-12

**Authors:** A. K. Tiwari, M. B. Mandal, S. B. Deshpande

**Affiliations:** Department of Physiology, Institute of Medical Sciences, Banaras Hindu University, Varanasi - 221005, India

**Keywords:** Gastric fundus contractions, *Mesobuthus tamulus* venom, serotonin, serotonergic mechanisms

## Abstract

**Aim:**

Gastric dysfunctions are commonly seen after scorpion envenomation, and the underlying mechanisms are not clear. Therefore, the present study was undertaken to investigate the effect of Indian red scorpion (*Mesobuthus tamulus, MBT*) venom on gastric fundus muscle contraction and the underlying mechanisms involved.

**Materials and Methods:**

*In vitro* isometric contraction was recorded from gastric fundus muscle strips on a chart recorder. The tissue was exposed to different concentrations of serotonin or crude MBT venom. The contractile responses to venom were expressed as the percentage of maximum contraction produced by serotonin at the beginning of each experiment. The contractile responses to 1.0 μg/ml of crude MBT venom were ascertained in the absence or presence of serotonin antagonist, methysergide.

**Results:**

Serotonin produced concentration-dependent fundus contractions (0.004–4.0 μM), and maximum contractile response was observed at 4.0 μM of serotonin. Hence, the contractile response obtained at 4.0 μM of serotonin was taken for normalization. The crude MBT venom (0.1–1.0 μg/ml) produced a concentration-dependent increase in fundus contractions (as % of maximum fundus contraction produced by serotonin at 4.0 μM). The maximum response was observed at 1.0 μg/ml of crude venom and a further increase in the concentration, up to 3.0 μg/ml, did not increase the response. In a separate series of experiments, pre-treatment with methysergide (1.0 μM) significantly attenuated the contractile response elicited by the venom (1.0 μg/ml) (*P*<0.05) and blocked the serotonin (4.0 μM) response.

**Conclusion:**

The results suggest that the crude MBT venom produces gastric fundus contractions by partially involving serotonin.

## Introduction

Indian red scorpion *(Mesobuthus tamulus, MBT)* envenomation produces severe systemic toxicity resulting in morbidity and mortality.[1–4] Besides cardiopulmonary and neuronal complications, the gastrointestinal effects are the serious manifestations of envenomation.[1,2,5] In scorpion-stung cases, more than 73% of the cases manifest with gastrointestinal problems. The commonly observed problems are vomiting (>70%), nausea and dysphagia.[5] The above symptoms indicate abnormality in gastric motility. Studies elsewhere demonstrate that the scorpion venom alters gastric functions by causing gastric ischemia or by increasing the gastric secretions and ulcerations.[6,7] The gastric contractility has relevance to the altered gastric functions associated with scorpion stings.[5] But, the information about the effect of scorpion venom on gastric contractility in terms of increased or decreased contractions is lacking. Therefore, the present study was designed to investigate the effect of crude *MBT* venom on gastric fundus contractions and elucidate the underlying mechanism of action.

## Materials and Methods

The experiments were performed as per the guidelines laid down by the Institutional Animal Ethics Committee.

### Drugs and chemicals

Lyophilized crude MBT venom (Haffkine Institute, Mumbai, India), serotonin creatine sulfate (Sigma Chemicals Co., St. Louis, MO, USA) and methysergide maleate (Sandoz, New Jersey, NJ, USA) were used in the present study. The stock solutions of serotonin (10^−2^ M) and methysergide (5 × 10^−3^ M) were prepared in distilled water and subsequent dilutions were made with Tyrode solution at the time of experimentation. The composition of Tyrode solution (mM) was: NaCl 137, KCl 3.7, MgCl_2_ 0.05, CaCl_2_ 1.02, NaH_2_PO_4_ 0.32, NaHCO_3_ 11.9 and glucose 5.0. All the chemicals used in the study were of analytical grade.

### Recording of gastric fundus muscle contractions

The methods for preparation and recording of gastric fundus contractions were followed as described earlier.[8] Adult rats of the Charles-Foster strain (3–4 months old) were used in the study. Each animal was kept in a plastic cage (36 cm × 23.5 cm × 14 cm) in an airconditioned animal room (temperature = 25 ± 0.5°C) and all animals were exposed to a 12:12-h dark:light cycle. Food and water were available to the animals *ad libitum*. The animals fasted overnight (received only water *ad libitum*), were sacrificed by cervical dislocation and exsanguination. The upper abdominal region was approached through a ventral midline incision and the fundus portion of the stomach was excised and kept in a petridish containing Tyrode solution bubbled continuously with 100% O_2_. Two longitudinal muscle strips measuring 25 mm × 4 mm each were prepared from each gastric fundus. The muscle strip was mounted vertically in Dale's organ bath (containing 25 ml Tyrode solution bubbled continuously with 100% O_2_ at 33 ± 1°C) by attaching one end to the glass tube and the other end to a Statham strain gauge isometric force transducer via a piece of thread as described earlier.[8] The Tyrode solution was changed at intervals of 15 min unless mentioned otherwise.

The gastric fundus muscle strip was given an initial resting tension of 0.5 g and was allowed to equilibrate for 30 min. Evoked contractions to different concentrations (0.004–40.0 *μ*M) of serotonin were recorded. The tissue was exposed to a given concentration of serotonin for 1 min and a minimum of 5 min was allowed between two exposures (after washing the preceding concentration twice). The area under the curve for 1 min was computed. The time-response area produced by serotonin was calculated.

The contractile responses of fundus muscle strips to different concentrations of MBT venom (0.1–3.0 *μ*g/ml) were recorded for 1 minute and the time-response area was computed. A given fundus strip was exposed to a single concentration of crude venom. The time-response area was expressed as % of maximum contractile response produced by serotonin (4.0 *μ*M).

In another series of experiments (n = 6 preparations), after obtaining the maximum contractile response to serotonin (4.0 *μ*M), the gastric fundus contractions to MBT venom (1.0 *μ*g/ml) in the absence or presence of methysergide (1.0 *μ*M for 30 min) were recorded. The time-response area was computed and expressed as % of maximum serotonin response as described before.

### Statistical analysis

The data are expressed as mean ± SEM. One way analysis of variance (ANOVA) was used to test the significance of responses obtained with various concentrations of serotonin/crude venom. Statistical comparisons of two groups were performed using Students t-test for paired or unpaired observations wherever applicable. A *P*-value <0.05 was considered significant.

## Results

### Serotonin produced gastric fundus muscle contractions

Serotonin produced a concentration-dependent increase in the contractions (time-response area) from 0.004 to 4.0 *μ*M (n = 6). Maximum contractile response was observed at 4.0 *μ*M, as 10-times increase in the concentration of serotonin (40.0 *μ*M) failed to increase the magnitude of the response any further [Table 1]. Hence, the response obtained at 4.0 *μ*M of serotonin was taken as the maximum response and was used to normalize the responses induced by crude MBT venom. The concentration of serotonin required to produce a half maximal response (EC50) was 0.085 ± 0.015 *μ*M (pD2 = 1.08).

**Table 1:** Effects of serotonin on gastric fundus muscle contractions

*Serotonin (*μ*M)*	*Gastric fundus contractility (% of maximum serotonin response)*
0.004	10.0 ± 1.0
0.04	27.0 ± 4.0
0.4	60.0 ± 5.0
4.0	96.0 ± 2.5
40.0	85.0 ± 6.5

The mean ± SEM values of gastric muscle contractile responses to different concentrations of serotonin are shown (n = 6 preparations at each concentration). The responses were expressed as % of maximum serotonin responses. The data reveal maximum fundus contractility at 4 μM of serotonin. The responses at each concentration are significantly different from one another (*P*<0.05, one-way ANOVA)

### Crude MBT venom-induced gastric fundus contractions

MBT venom produced a concentration-dependent (0.1–1.0 *μ*g/ml) increase in gastric fundus muscle contractions. The maximum response was seen with 1.0 *μ*g /ml of venom. Further increase in the concentration of venom (3.0 *μ*g /ml) did not increase the response any further [Table 2]. The EC50 concentration was 0.48 ± 0.07 *μ*g/ml (pD2 = 0.318).

**Table 2:** Effects of crude MBT venom on gastric fundus muscle contractions

*MBT venom (*μ*g/ml)*	*Gastric fundus contractility (% of maximum serotonin response)*
0.1	2.0 ± 0.50
0.3	12.7 ± 2.51
0.6	27.8 ± 4.52
1.0	45.8 ± 6.77
3.0	35.2 ± 6.15

The mean ± SEM values of gastric muscle contractile responses (time–response area) to different concentrations of crude MBT venom are shown (n = 6 preparations at each concentration). The responses were expressed as % of maximum serotonin responses. The responses at each concentration are significantly different from one another (*P*<0.05, one-way ANOVA)

### MBT venom-induced fundus contractions were antagonized by methysergide

The contractile responses to crude MBT venom (1.0 *μ*g/ml) without exposure to methysergide in this set of experiments were 43.76 ± 6.33 (% of maximum response obtained with serotonin [4.0 *μ*M; n = 6 preparations]), whereas in the methysergide (1.0 *μ*M for 30 min)-pre-treated group (n = 6 preparations), responses were reduced to 16.67 ± 5.36 (as % of maximum response obtained with serotonin). The contractile responses to venom in the presence of methysergide were significantly different as compared with control responses (*P* <0.05, Student's t-test for unpaired observations). However, in the methysergide (1.0 *μ*M)-pre-treated group, the response to 4.0 *μ*M of serotonin was completely blocked.

## Discussion

The results of this study demonstrated that crude MBT venom produced gastric fundus contractions and that these contractions were mediated by serotonergic mechanisms.

MBT venom increased gastric fundus contractions in a concentration-dependent (0.1–1.0 *μ*g/ml) manner [Table 2]. The maximum contraction was observed with 1.0 *μ*g/ml of MBT venom. However, a further increase in the concentration of venom (3.0 *μ*g/ml) did not enhance the contractility any further. Reports elsewhere suggest that venom mediates its gastrointestinal effects by causing liberation of a massive amount of neurotransmitters.[5] Further, serotonin has been reported to be present in high concentration in enterochromaffin cells (EC) and myenteric plexuses of the stomach.[9] Therefore, venom may induce gastric contractions by liberating serotonin from EC cells or myenteric plexuses. Further, in this study, pre-treatment of the gastric fundus muscle tissue with a serotonin receptor antagonist, methysergide (1.0 *μ*M), blocked the MBT (1.0 *μ*g/ml) venom-induced contractility (*P* 0.05). This indicates the involvement of serotonergic mechanisms in MBT venom-induced gastric fundus muscle contractions.

Serotonin is shown to be present in scorpion venom from many species,[10–13] but the concentration of serotonin present in 1 *μ*g/ml of crude MBT venom is too low (< 0.0001 *μ*M)[10] to account for the magnitude of contractile response observed in this study [Table 2]. Thus, the above results not only support the action of serotonin but also indicate the role of other constituents/mechanisms of crude venom in mediating fundus muscle contractions.

The partial blockade of venom-induced fundus muscle contractile response by methysergide (62%) suggests the involvement of other non-serotonergic mechanisms in mediating the contractions. These mechanisms include toxins, histamine and kinins and are reported to be activated in envenomed animals.[12–19] Reports also indicate that the crude venom mediates its actions via histamine/kinin mechanisms.[1,2,12,13,17–19] Further experiments are necessary to explore non-serotonergic mechanisms that may help in the understanding of venom-induced gastric contractions.

## Conclusion

Results of this study suggest that the crude MBT venom produces gastric fundus contractions that are partially, but predominantly, mediated by serotonergic mechanisms.
